# Canadian dentists' views on the first dental visit for children

**DOI:** 10.3389/froh.2022.957205

**Published:** 2022-08-25

**Authors:** Hamideh Alai-Towfigh, Robert J. Schroth, Ralph Hu, Victor H. K. Lee, Olubukola Olatosi

**Affiliations:** ^1^Department of Preventive Dental Science, Dr. Gerald Niznick College of Dentistry, Rady Faculty of Health Sciences, University of Manitoba, Winnipeg, MB, Canada; ^2^Children's Hospital Research Institute of Manitoba, Winnipeg, MB, Canada; ^3^Departments of Pediatrics and Child Health and Community Health Sciences, Max Rady College of Medicine, Rady Faculty of Health Sciences, University of Manitoba, Winnipeg, MB, Canada; ^4^Shared Health Inc., Winnipeg, MB, Canada; ^5^Department of Oral Biology, Dr. Gerald Niznick College of Dentistry, Rady Faculty of Health Sciences, University of Manitoba, Winnipeg, MB, Canada

**Keywords:** first dental visit, access to care, health knowledge, attitudes, practice, preventive dentistry, pediatric dentistry, early childhood caries

## Abstract

**Introduction:**

Early dental visits set children on an upward trajectory, toward a lifetime of optimal oral health. The purpose of this study was to analyze data from a survey of Canadian dentists to determine their knowledge, attitudes, and behaviors regarding first dental visits.

**Methods:**

The Canadian Dental Association (CDA) surveyed general and pediatric dentists regarding the timing of the first dental visit. Demographic and practice information was collected. Analyses included descriptive analyses, bivariate analyses, and multiple logistic regression with forward stepwise selection. Significance was set at *p* ≤ 0.05.

**Results:**

Overall, 3,232 dentists participated. The majority were male (58.5%), general dentists (96.6%), in non-metropolitan areas (50.5%), and practiced for 20.6 ± 12.8 years. The mean age recommended for first visits was 20.4 ± 10.8 months. Only 45.4% of dentists recommended a first visit ≤ 12 months. A majority (59.5%) knew that the correct age recommended for first visits was no later than 12 months. Most dentists who had seen a patient ≤ 12 months before did not typically do so (82.3%). General dentists were 61% less likely to recommend first visits by 12 months (OR = 0.39; 95% CI: 0.16, 0.91). Dentists in Central Canada (OR = 1.83; 95% CI: 1.44, 2.32); dentists who typically saw patients ≤ 12 months (OR = 3.41; 95% CI: 2.41, 4.83); those who echoed the importance of visits by 12 months (OR = 19.3; 95% CI: 8.2, 45.71); dentists with staff that encouraged infant/toddler care (OR = 1.76; 95% CI: 1.34, 2.31); and those who knew official North American recommendations for first visits (OR = 5.28; 95% CI: 4.13, 6.76) were all more likely to recommend first visits by 12 months.

**Conclusions:**

A majority of Canadian dentists did not recommend first visits by 12 months of age, despite it being the CDA's official position. Provider characteristics can influence the age that is recommended for first visits. Findings from this study may inform educational campaigns on early childhood oral health targeted toward dentists.

## Introduction

Early childhood caries (ECC) is a highly prevalent public health issue worldwide. Approximately 24% of children younger than 36 months and 57% of children between the ages of 3–6 years suffer from ECC [1]. The prevalence of ECC is as high as 90% in some parts of Canada [2]. If left untreated, ECC can negatively impact a child's overall well-being by causing pain, behavioral problems, difficulties eating, speech problems, impediments in learning, and a decrease in oral health-related quality of life [3]. Preventive approaches are preferred over the surgical treatment of disease in children [4]. However, dental surgery to treat severe ECC is known as the most common surgical procedure in preschool children at most Canadian pediatric and community hospitals [5].

Early first dental visits may be protective against ECC, as dentists can identify high-risk children before significant problems arise [4, 6, 7]. Current established professional organizations recommend a first visit no later than 12 months of age [5]. Unfortunately, early first dental visits are atypical [8, 9]. A recent study reported that <1% of healthy, urban, Canadian children visit the dentist by age one, and about 2% of children visited the dentist by age two [5, 9]. Establishing a dental home by age one is encouraged and has proven to be effective [5, 8, 10]. This can help parents or caregivers develop proper oral health habits early in their child's life, rather than trying to change unhealthy habits later on [11]. Early preventive dental care can reduce the need for future restorative appointments and visits to the emergency room, while also decreasing associated costs [9, 10, 12].

Earlier appointments may be more common in certain regions of Canada where there have been campaigns promoting early visits [13]. These first dental visits set children on an upward trajectory, toward a lifetime of optimal oral health. The concept may seem new to some, but in 1986, the American Academy of Pediatric Dentistry (AAPD) first published “Infant Oral Health Guidelines” and recommended an oral examination and assessment within six months of the eruption of the first tooth and no later than 12 months of age [6]. This recommendation is 35 years old, but it is likely not known by all practicing dentists. The Canadian Dental Association (CDA) also endorses a first visit by 12 months of age [5].

No national data has been published on the views and attitudes of Canadian dentists on early childhood dental visits. This is information is only available for specific regions. This study assessed the knowledge, attitudes, and behaviors of dentists in Canada regarding the timing of the first dental visit and the importance of developing a positive relationship between the child, family, and dental team.

## Methods

In 2013, the CDA undertook a national survey of dentists. The survey covered first dental visits, as this was one of two priority areas identified by the CDA's Access to Care Working Group. General and pediatric dentists received email invitations to complete an electronic survey, which collected demographic and practice information. The survey covered several topics, including dentists' awareness and knowledge of infant and toddler dental care, timing of the first dental visit, knowledge of professional recommendations on first dental visits, and views on ECC.

The online survey was administered by Navigator Ltd., which was contracted by the CDA. The survey was e-mailed on January 2013 to 14,747 general and pediatric dentists. To increase the number of respondents, two follow-up emails were sent. Specific objectives of the survey were to (1) determine the average recommended age for a first dental visit by Canadian dentists, (2) determine which factors and provider characteristics were associated with earlier recommended first dental visits, and (3) inform the CDA's advocacy efforts to promote young children's oral health.

The CDA provided approval for the secondary analysis of the survey data. Ethics approval was also obtained from the University of Manitoba's Health Research Ethics Board. The key outcome variable was the age dentists recommended for a first dental visit, and the proportion who recommended first visit ≤ 12 months. Several other variables of interest were also considered, including gender, year of graduation, type of dentist, and type of practice. The key outcome was dichotomized into those who recommended a first visit ≤ 12 months of age, and those recommending first visits >12 months of age. Provinces and territories were grouped into Western (Alberta, British Columbia, Manitoba, Northwest Territories, Nunavut, Saskatchewan, Yukon,); Central (Ontario, Quebec); and Eastern Canada (New Brunswick, Newfoundland and Labrador, Nova Scotia, Prince Edward Island). Practice location was coded as being in a census metropolitan or non-census metropolitan area (census metropolitan defined as a total population of ≥100,000, with ≥50,000 in the urban core; non-census metropolitan areas are smaller urban areas with a population <100,000). The types of practice were recoded as solo, group, or non-private practices.

Data were analyzed using Number Cruncher Statistical Software (Version 20.0.2; Kaysville, Utah). Descriptive statistics [means, standard deviations (SD), and frequencies] were calculated. Data was analyzed comparing general dentists vs. pediatric dentists and the recommendation of first dental visit ≤ 12 months vs. >12 months. Relationships between participant characteristics and age of first visit recommended by dentists were evaluated by Chi-square for categorical variables, and *t*-tests and analysis of variance (ANOVA) for continuous variables. Correlation models were used to examine the dependent variables of number of years in practice and age that dentists recommend for first visit. Multiple logistic regression with forward stepwise selection was used for the key outcome of recommending first visits by 12 months of age. A *p*-value of ≤ 0.05 was considered significant.

## Results

A total of 3,232 dentists participated in the study (response rate of 21.9%). General characteristics are highlighted in Table 1. A majority of participants were male (58.5%), general dentists (96.6%), working in group private practices (51.7%), living in non-census metropolitan areas (50.5%), and were from Ontario (42.6%). Dentists practiced for an average of 20.6 ± 12.8 years.

**Table 1:** Participant characteristics.

**Variable**	***N* (%)**
**Province/territory:**
Alberta	401 (12.4)
British Columbia	538 (16.6)
Manitoba	129 (4.0)
New Brunswick	222 (6.9)
Newfoundland and Labrador	83 (2.6)
Nova Scotia	44 (1.4)
Nunavut	2 (0.1)
Northwest Territories	2 (0.1)
Ontario	1,382 (42.6)
Prince Edward Island	32 (1.0)
Quebec	214 (6.6)
Saskatchewan	174 (5.4)
Yukon	9 (0.3)
**Location in Canada:**
Central Canada	1,596 (49.4)
Western Canada	125S5 (38.8)
Eastern Canada	381 (11.8)
**Location of practice:**
Census metropolitan	1,586 (49.5)
Non-census metropolitan	1,621 (50.5)
**Gender:**
Male	1,889 (58.5)
Female	1,343 (41.5)
**Year of graduation:**
1951–1970	123 (4.0)
1971–1980	506 (16.4)
1981–1990	790 (25.5)
1991–2000	675 (21.8)
2001–2013	1,007 (32.3)
**Years in practice (mean** **±SD)**	20.6 ± 12.8
**Type of dentist:**
General dentist	3,122 (96.6)
Pediatric dentist	110 (3.4)
**Type of practice:**
Group private practice	1,671 (51.7)
Solo private practice	1,355 (41.9)
Non-private practice	206 (6.4)

Table 2 highlights participants' knowledge, attitudes, and behaviors regarding first dental visits. Approximately 45.2% of respondents actually recommended a first visit by 12 months of age. About 60% of respondents believed that dental organizations recommended first visits as soon as the first primary tooth erupts; 64.8% of dentists were aware of the CDA's position on first dental visits. While a majority of participants (74.2%) had seen a patient under 12 months of age before, this was atypical, with dentists frequently seeing older children for the first time (82.3%). Those that did not typically see children under 12 months often referred younger children to colleagues (55.3%). The majority (76.0%) of respondents felt that parents did not understand the importance of a child's first visit to a dentist, and only 14% felt that parents or caregivers were open to bringing their infant and/or toddler to the dentist before 12 months of age.

**Table 2:** Participants' knowledge, attitudes, and behaviors about the first dental visit.

**Variable**	***N* (%)**
**Age recommended for a child's first dental visit (months):**	
0–6 months	242 (7.5)
7–12 months	1,206 (37.7)
13–24 months	829 (25.9)
25–36 months	860 (26.9)
37–48 months	57 (1.8)
49–72 months	8 (0.2)
**Age dentists believe dental organizations in North America recommend** **a first dental visit:**	
As soon as the first tooth erupts and no later than 12 months	1,863 (59.5)
Between 1–2 years	578 (18.5)
After 2 years and before attending pre-school	130 (4.2)
At 3 years	316 (10.1)
Don't know	244 (7.8)
**Ever seen a patient** **≤12 months of age:**	
Yes	2,324 (74.2)
No	807 (25.8)
**Typically see patients** **≤12 months of age:**	
Yes	554 (17.7)
No	2,577 (82.3)
**Does not see patients** **≤12 months of age, but refer to a colleague who does:**	
Yes	1,303 (55.3)
No	1,052 (44.7)
**Actively discusses early childhood dental care with patients:**	
Yes	2,694 (94.8)
No	149 (5.2)
**Provide parents of infants and toddlers with information on how to care** **for their child's teeth:**	
Yes	2,688 (94.5)
No	155 (5.5)
**Promotes early visits for infants and toddlers in your practice:**	
Yes	2,246 (79.0)
No	597 (21.0)
**Uses “knee to knee positioning” when examining infants and toddlers:**	
Yes	1,659 (61.0)
No	1,059 (39.0)
**After examining an infant and/or toddler for the first time, typically** **suggests that the child returns for their next visit…**	
Within 6 months	1,052 (38.7)
Within 1 year	464 (17.1)
After 1 year	98 (3.6)
I do not recommend when a child should return	29 (1.1)
Depends on their risk for caries (caries-risk assessment)	1,075 (39.5)
**Parents are open to the idea of bringing their infant/toddler in for an** **examination before 12 months of age:**	
Very open	379 (14.0)
Neutral	761 (28.0)
Not at all open	330 (12.1)
Don't know	533 (19.6)
Interested with appropriate guidance	715 (26.3)
**Parents adequately understand the importance of a child's first visit to a** **dentist?**	
Yes	651 (24.0)
No	2,067 (76.0)
**Important for a child to receive their first dental examination within the** **first 6 months of the eruption of the first tooth, or by 1 year of age:**	
Agree	1,313 (50.9)
Somewhat agree	583 (22.5)
Neither agree nor disagree	355 (13.7)
Somewhat disagree	218 (8.4)
Disagree	116 (4.5)
**Confident in my ability to perform a dental examination on an infant:**	
Agree	1,419 (55.0)
Somewhat agree	701 (27.1)
Neither agree nor disagree	251 (9.7)
Somewhat disagree	158 (6.1)
Disagree	55 (2.1)
**Confident in my ability to perform a dental examination on a toddler:**	
Agree	1,728 (67.6)
Somewhat agree	604 (23.4)
Neither agree nor disagree	138 (5.3)
Somewhat disagree	77 (2.3)
Disagree	37 (1.4)
**Before feeling comfortable treating an infant or toddler, would require** **more training:**	
Agree	327 (12.7)
Somewhat agree	543 (21.0)
Neither agree nor disagree	659 (25.5)
Somewhat disagree	429 (16.6)
Disagree	626 (24.2)
**Front office staff actively encourages infant and toddler dental care:**	
Agree	758 (29.4)
Somewhat agree	690 (26.7)
Neither agree nor disagree	714 (27.6)
Somewhat disagree	275 (10.6)
Disagree	147 (5.7)
**Staff is comfortable dealing with infants and toddlers in our dental** **practice:**	
Agree	1,025 (39.6)
Somewhat agree	878 (34.0)
Neither agree nor disagree	418 (16.2)
Somewhat disagree	194 (7.5)
Disagree	69 (2.7)
**Would like to receive additional training on how to incorporate early** **childhood care into my practice:**	
Agree	636 (24.7)
Somewhat agree	793 (30.7)
Neither agree nor disagree	629 (24.3)
Somewhat disagree	241 (9.3)
Disagree	285 (11.0)
**Aware of CDA's position on first dental visit:**	
Yes	1,634 (64.8)
No	888 (35.2)

A majority of dentists agreed (50.9%) or somewhat agreed (22.5%) that it is important for a child to receive their first dental examination within 6 months of the eruption of the first tooth, or by 12 months of age. Furthermore, most dentists expressed confidence in their ability to perform dental examinations on infants and toddlers. A third of dentists (33.7%) agreed or somewhat agreed that they would require additional training before they felt comfortable treating infants and toddlers.

**Figure 1:**
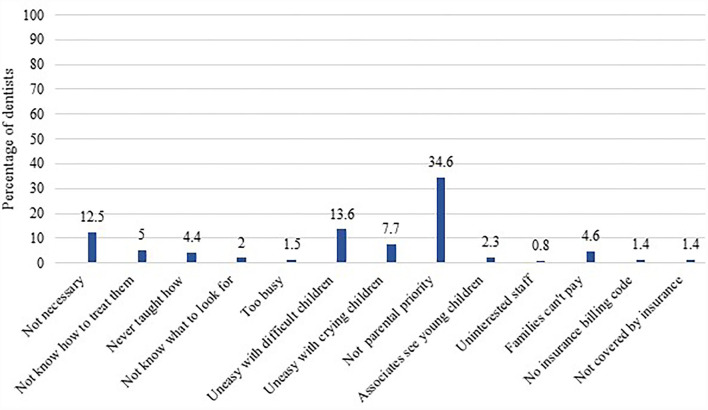
Reasons participating dentists gave for not seeing patients ≤ 12 months of age.

**Figure 2:**
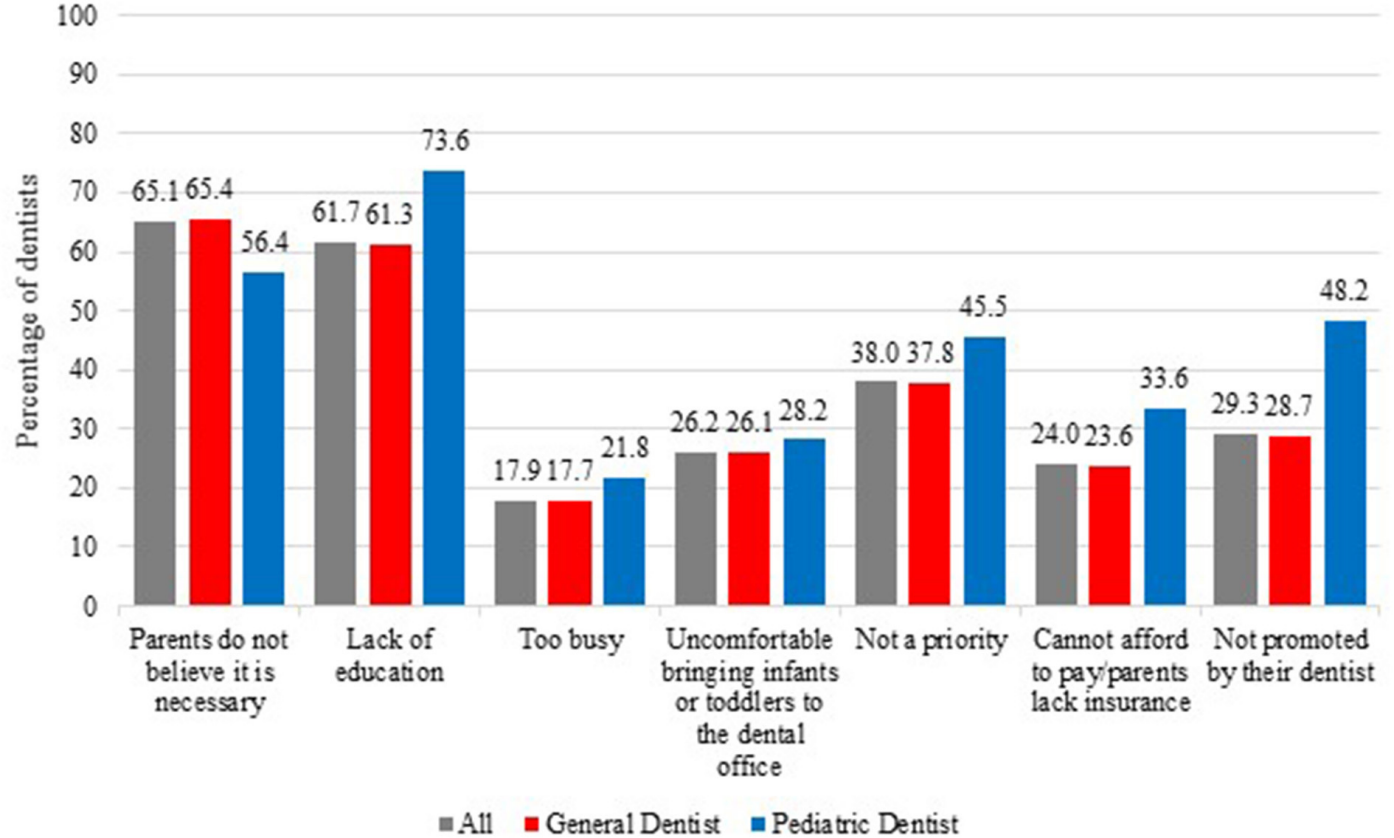
Reasons dentists gave as to why parents or caregivers did not bring infants/toddlers for early first visits.

The three most common reasons given by dentists for not seeing patients ≤ 12 months of age were that they believed that parents did not see it as a priority (34.6%), they were uncomfortable seeing difficult children (13.6%), and they felt it was not necessary to see children of that age range (12.5%; Figure 1). The three most common reasons given by dentists for parents or caregivers not bringing their child to the dentist within the first year of life were that they believed that parents did not think it was necessary (65.1%), they believed that parents lacked education and awareness (61.7%), and they believed that parents did not see it as a priority for their child (38%; Figure 2).

Table 3 highlights respondent characteristics, as they relate to visit recommendations (i.e., ≤ 12 months or >12 months) and the mean recommended age for first visits. The Northwest Territories (100%), Manitoba (62.5%), New Brunswick (61.8%), and British Columbia (60.8%) had the highest percentages of dentists recommending the correct age for first visits (*p* < 0.0001). These regions also had the lowest mean recommended ages for first visits (*p* < 0.0001).

**Table 3:** Association between respondent characteristics and recommended age of first visit.

**Variables**	**Mean age recommended (months)**	***p-*Value**	** ≤ 12 months**	**>12 months**	***p-*Value**
**Province/territory:**
Alberta	20.2 ± 10.3	<0.0001^c^	169 (42.8)	226 (57.2)	<0.0001^a^
British Columbia	16.8 ± 9.1		322 (60.8)	208 (39.3)	
Manitoba	17.2 ± 10.6		80 (62.5)	48 (37.5)	
New Brunswick	16.8 ± 9.1		136 (61.8)	84 (38.2)	
Newfoundland and Labrador	23.0 ± 10.7		29 (35.4)	53 (64.6)	
Nova Scotia	19.0 ± 8.5		19 (43.2)	25(56.8)	
Nunavut	21.0 ± 21.2		1 (50.0)	1 (50.0)	
Northwest Territories	12.0 ± 0.0		2 (100.0)	0 (0.0)	
Ontario	22.2 ± 11.2		528 (38.5)	843 (61.5)	
Prince Edward Island	23.7 ± 12.4		12 (37.5)	20 (62.5)	
Quebec	22.4 ±11.1		78 (37.0)	133 (63.0)	
Saskatchewan	21.4 ± 10.2		74 (42.5)	100 (57.5)	
Yukon	19.1 ± 7.3		3 (33.3)	6 (66.7)	
**Location of practice:**
Census metropolitan	20.3 ± 10.7	0.62^b^	720 (45.6)	860 (54.4)	0.85^a^
Non-census metropolitan	20.5 ± 10.8		725 (45.2)	878 (54.8)	
**Gender:**
Male	22.2 ± 10.9	<0.0001^b^	699 (37.4)	1,171 (62.6)	<0.0001^a^
Female	18.0 ± 10.0		749(56.2)	583 (43.8)	
**Years in practice**	*r* = 0.33	<0.0001^d^	16.2 ± 12.5	24.2 ± 11.9	<0.0001^b^
**Type of dentist:**
General dentist	20.7 ± 10.8	<0.0001^b^	1,353 (43.8)	1,739 (56.2)	<0.0001^a^
Pediatric dentist	12.6 ± 5.2		95 (86.4)	15 (13.6)	
**Type of practice:**
Solo private practice	21.7 ± 10.9	<0.0001^c^	519 (38.6)	824 (61.4)	<0.0001^a^
Group private practice	20.0 ± 10.7		794(47.9)	863 (52.1)	
Non-private practice	15.7 ± 8.9		135 (66.8)	67 (33.2)	

a Chi-square test.

b t-test.

c ANOVA.

d Correlation analyses.

Female dentists were significantly more likely to recommend first visits ≤ 12 months of age than their male colleagues (56.2% vs. 37.4%, *p* < 0.0001; Table 3). The mean ages for first visits recommended by female dentists were also significantly lower than their male colleagues (18.0 ± 10 months vs. 22.2 ± 10.9 months, *p* < 0.0001). Dentists who recommended a first visit by 12 months of age practiced for significantly fewer years than those who recommended a first visit past 12 months (16.2 ± 12.5 vs. 24.2 ± 11.9, *p* < 0.0001). A greater proportion of pediatric dentists (86.4%), compared to general dentists (43.8%) recommended a first visit ≤ 12 months of age (*p* < 0.0001). Pediatric dentists tended to recommend first visits earlier on, while general dentists provided later suggestions (12.6 ± 5.2 months vs. 20.7 ± 10.8, *p* < 0.0001). The mean ages for first visits recommended by general dentists in non-private practices (e.g., community-, hospital-, or university-based; 15.7 ± 9.0) were closer to the correct age, than those suggestions given by dentists in solo (21.7 ± 10.9) or group practices (20.0 ± 10.7, *p* < 0.0001).

Table 4 reports on respondents' recommendations of first dental visits in relation to dentists' knowledge, attitudes, behaviors. Dentists were dichotomized as to whether they were recommending a first visit ≤ 12 months of age or >12 months. Overall, 63.8% of dentists who knew the age that dental organizations in North America recommended for first visits utilized those recommendations in practice themselves. Dentists who typically saw patients <12 months (84%) were more likely to recommend first visits ≤ 12 months over those that did not typically see patients of that age range (37.4%; *p* < 0.0001). Dentists who used “knee-to-knee positioning” were also more likely to recommend visits ≤ 12 months over those that did not use the technique (54.8% vs. 28.2%, *p* < 0.0001). The majority of dentists who agreed it was important for a child to receive their first dental examination within the first 6 months of the eruption of the first tooth, or by 1 year of age (76.9%), tended to recommended visits ≤ 12 months for first visit (*p* < 0.0001).

**Table 4:** Associations between knowledge, attitudes, behaviors, mean age recommended, and actual recommendations by 12 months of age.

**Variables**	**Mean age recommended (months)**	***p-*Value**	** ≤ 12 months**	**>12 months**	***p-*Value**
**Age dentists believe that dental organizations in North America recommend a first dental visit:**					
As soon as the first tooth erupts and no later than 12 months	16.5 ± 9.5	<0.0001	1,184 (63.8)	671 (36.2)	<0.0001
Between 1 and 2 years	21.6 ± 8.5		162 (28.2)	412 (71.8)	
After 2 years and before attending pre-school	28.9 ± 8.0		5 (3.9)	124 (96.1)	
At 3 years	31.6 ± 9.3		29 (9.5)	278 (90.6)	
Don't know	28.0 ± 10.4		37 (15.4)	203 (84.6)	
**Ever seen a patient** **≤12 months for an infant/toddler visit:**					
Yes	19.3 ± 10.5	<0.0001	1,149 (49.8)	1,160 (50.2)	<0.0001
No	23.5 ± 10.8		268 (33.7)	528 (66.3)	
**Typically sees a patient** **≤12 months of age:**					
Yes	12.3 ± 7.0	<0.0001	462 (84.0)	88 (16.0)	<0.0001
No	22.1 ± 10.6		955 (37.4)	1,600 (62.6)	
**Does not see patients** **≤12 months of age, but refers to a colleague who does:**					
Yes	22.2 ± 10.9	>0.05	485 (37.6)	806 (62.4)	0.0067
No	22.9 ± 10.3		336 (32.2)	708 (67.8)	
**Actively discusses early childhood dental care with patients:**					
Yes	20.5 ± 10.8	<0.001	1,203 (44.8)	1,480 (55.2)	0.0014
No	23.8 ± 11.5		45 (31.3)	99 (68.8)	
**Promotes early visits for infants and toddlers in your practice:**					
Yes	18.2 ± 9.7	<0.0001	1,168 (52.2)	1,068 (47.8)	<0.0001
No	29.9 ± 9.8		80 (13.5)	511 (86.5)	
**Uses “knee-to-knee” positioning when examining infants and toddlers:**					
Yes	18.2 ± 10.0	<0.0001	907 (54.8)	748 (45.2)	<0.0001
No	24.4 ± 10.9		296 (28.2)	753 (71.8)	
**Important for a child to receive their first dental examination within the first 6 months of the eruption of the first tooth, or by 1 year of age:**					
Agree	13.6 ± 7.3	<0.0001	1,006 (76.9)	302 (23.1)	<0.0001
Somewhat agree	23.4 ± 9.3		132 (22.8)	447 (77.2)	
Neither agree nor disagree	29.5 ± 7.8		19 (5.4)	333 (94.6)	
Somewhat disagree	31.1 ± 7.1		5 (2.3)	213 (97.7)	
Disagree	33.3 ± 7.2		2 (1.7)	114 (98.3)	
**Confident in my ability to perform a dental examination on an infant:**					
Agree	17.8 ± 10.0	<0.0001	789 (55.8)	624 (44.2)	<0.0001
Somewhat agree	22.0 ± 10.5		264 (37.7)	435 (62.3)	
Neither agree nor disagree	24.4 ± 10.5		72 (29.0)	176 (71.0)	
Somewhat disagree	26.5 ± 10.2		35 (21.5)	123 (78.5)	
Disagree	30.3 ± 6.1		5 (9.3)	49 (90.7)	
**Confident in my ability to perform a dental examination on a toddler:**					
Agree	18.8 ± 10.3	<0.0001	876 (50.8)	847 (49.2)	<0.0001
Somewhat agree	22.6 ± 10.5		213 (35.3)	390 (64.7)	
Neither agree nor disagree	24.5 ± 11.6		47 (35.6)	89 (65.4)	
Somewhat disagree	24.6 ± 11.1		24 (31.6)	52 (68.4)	
Disagree	30.8 ± 11.0		4 (11.4)	31 (88.6)	
**Front office staff actively encourages infant and toddler dental care:**					
Agree	15.4 ± 8.7	<0.0001	507 (67.2)	247 (32.8)	<0.0001
Somewhat agree	20.1 ± 10.1		298 (43.2)	391 (56.8)	
Neither agree nor disagree	22.4 ± 10.6		254 (35.7)	458 (64.3)	
Somewhat disagree	26.0 ± 11.0		74 (27.0)	201 (73.0)	
Disagree	27.3 ± 11.3		32 (22.2)	112 (77.8)	
**Staff comfortable dealing with infants and toddlers in our dental practice:**					
Agree	17.5 ± 9.9	<0.0001	582 (57.1)	438 (42.9)	<0.0001
Somewhat agree	21.2 ± 10.2		345 (39.3)	532 (60.7)	
Neither agree nor disagree	23.1 ± 11.0		151 (36.4)	264 (63.6)	
Somewhat disagree	24.4 ± 11.7		63 (32.5)	131 (67.5)	
Disagree	24.6 ± 12.7		23 (34.3)	44 (65.7)	
**Aware of CDA's position on first dental visit:**
Yes	18.3 ± 10.1	<0.0001	890 (54.6)	740 (45.4)	<0.0001
No	24.3 ± 10.8		244 (27.7)	638 (72.3)	
**Does not see patients under 12 months because**…..
i) It is not necessary:					
Yes	29.7 ± 8.0	<0.0001	26 (6.4)	378 (93.6)	<0.0001
No	19.1 ± 10.4		1,422 (50.8)	1,376 (49.2)	
ii) Do not know how to treat them:					
Yes	27.0 ± 10.6	<0.0001	34 (21.0)	128 (79.0)	<0.0001
No	20.1 ± 10.7		1,414 (46.5)	1,626 (53.5)	
iii) Was never taught how:					
Yes	28.3 ± 9.8	<0.0001	22 (15.8)	117 (84.2)	<0.0001
No	20.1 ± 10.7		1,426 (46.6)	1,637 (53.4)	
iv) Do not know what to look for:					
Yes	27.4 ± 10.0	<0.0001	13 (19.4)	54 (80.6)	<0.0001
No	20.3 ± 10.7		1,435 (45.8)	1,700 (54.2)	
v) Too busy:					
Yes	26.8 ± 10.9	<0.001	9 (18.8)	39 (81.3)	0.0002
No	20.3 ± 10.7		1,439 (45.6)	1,715 (54.4)	
vi) Uncomfortable seeing uncooperative children:					
Yes	25.7 ± 10.8	<0.0001	102 (23.2)	337 (76.8)	<0.0001
No	19.6 ± 10.5		1,346 (48.7)	1,417 (51.3)	
vii) Uncomfortable seeing crying children:					
Yes	26.9 ± 11.3	<0.0001	54 (21.6)	196 (78.4)	<0.0001
No	19.9 ± 10.5		1,394 (47.2)	1,558 (52.8)	
viii) Few parents see it as a priority and little demand from the public:					
Yes	21.9 ± 10.3	<0.0001	401 (35.9)	715 (64.1)	<0.0001
No	19.6 ± 10.9		1,047 (50.2)	1,039 (49.8)	
ix) Associates in my office see them instead:					
Yes	24.8 ± 12.0	0.0016	24 (32.4)	50 (67.6)	0.025
No	20.3 ± 10.7		1,424 (45.5)	1,704 (54.5)	
x) Staff not interested or supportive of seeing young children:					
Yes	22.8 ± 11.3	0.15	10 (38.5)	16 (61.5)	0.49
No	20.4 ± 10.8		1,438 (45.3)	1,738(54.7)	

Participants who recommended visits ≤ 12 months reported more confidence in their ability to perform exams on infants and toddler than those who recommend first visit >12 months, (55.8% vs. 44.2%, *p* < 0.0001; and 50.8% vs. 49.2%, *p* < 0.0001, respectively). The dentists who recommended first visits ≤ 12 months agreed that their front staff actively encouraged infant and toddler dental care (67.2%), and were comfortable dealing with infant and toddlers (57.1%). More of the dentists who recommended first visits ≤ 12 months were aware of CDA's position on first dental visits compared to those who recommended first visit >12 months (54.6 vs. 45.4%, *p* < 0.0001).

When comparing dentist types with other participant characteristics, more pediatric dentists practiced in census metropolitan areas compared to general dentists (87.2 vs. 48.1%, *p* < 0.0001) (Table 5). Pediatric dentists had also practiced for a longer amount of time than general dentists (23.2 ± 11.7 years vs. 20.6 ± 12.8, *p* < 0.0001). They were more likely to be in non-private practices, such as university- or hospital-based practices, than general dentists (18% vs. 6%, *p* < 0.0001). Pediatric dentists recommended first dental visits closer to the correct age (in months) compared to general dentists (12.6 ± 5.2 vs. 20.5 ± 10.8, *p* < 0.0001). More pediatric dentists knew the correct age that dental organizations were recommending for first visits compared to general dentists (81.5% vs. 58.7%, *p* < 0.0001). Compared to general dentists, pediatric dentists typically saw more patients under 12 months (66.7 vs. 16%, *p* < 0.0001), used “knee-to- knee positioning” more often (87 vs. 60.1%, *p* < 0.0001), and were more confident in seeing infants (96.6 vs. 53.4%, *p* < 0.0001). Pediatric dentists' staff were also extremely comfortable in dealing with infants and toddlers compared to the staff of general dentists (92.1 vs. 37.8%, *p* < 0.0001).

**Table 5:** Association between participant characteristics and type of dentist.

**Variables**	**General dentist**	**Pediatric dentist**	***p-*Value**
	***N*** **(%)**	***N*** **(%)**	
**Province/territory:**			
Alberta	389 (12.5)	12 (11.0)	0.06
British Columbia	516 (16.5)	22 (20.0)	
Manitoba	126 (4.0)	3 (2.7)	
New Brunswick	219 (7.0)	3 (2.7)	
Newfoundland and Labrador	82 (2.6)	1 (0.9)	
Nova Scotia	44 (1.4)	0 (0.0)	
Nunavut	2 (0.1)	0 (0.0)	
Northwest Territories	2 (0.1)	0 (0.0)	
Ontario	1,328 (42.5)	54 (49.1)	
Prince Edward Island	32 (1.0)	0 (0.0)	
Quebec	200 (6.4)	14 (12.7)	
Saskatchewan	173 (5.5)	1 (0.9)	
Yukon	9 (0.4)	0 (0.0)	
**Location of practice:**			
Census metropolitan	1,491 (48.1)	95 (87.2)	<0.0001
Non-census metropolitan	1,607 (51.9)	14 (12.8)	
**Gender:**			
Male	1,825 (58.5)	64 (58.2)	0.95
Female	1,297 (41.5)	46 (41.8)	
**Year of graduation:**			
1951–1970	114 (3.8)	9 (8.4)	0.0024
1971–1980	481 (16.1)	25 (23.4)	
1981–1990	768 (25.7)	22 (20.6)	
1991–2000	649 (21.7)	26 (24.3)	
2001–2013	972 (32.6)	25 (23.4)	
**Years in practice (mean** **±SD)**	20.6 ± 12.8	23.2 ± 11.7	<0.0001
**Type of practice:**			
Group private practice	1,615 (51.7)	56 (51.0)	<0.0001
Solo private practice	1,321 (42.3)	34 (31.0)	
Non-private practice	186 (6.0)	20 (18.0)	
**Mean recommend a child should see a dentist (months** **±SD)**	20.4 ± 10.8	12.6 ± 5.2	<0.0001
**Recommended age that child should see a dentist:**			
≤ 12 months	1,353 (43.8)	95 (86.4)	<0.0001
>12 months	1,739 (56.2)	15 (13.6)	
**Age dentists believe that dental organizations in North America recommend a first dental visit:**			
As soon as the first tooth erupts and no later than 12 months	1,775 (58.7)	88 (59.5)	<0.0001
Between 1–2 years	567 (18.8)	11 (18.5)	
After 2 years and before attending pre-school	128 (4.2)	2 (4.1)	
At 3 years	311 (10.3)	5 (10.1)	
Don't know	242 (8.0)	2 (7.8)	
**Age dentists believe that dental organizations in North America recommend a first dental visit**:			
≤ 12 months	1,775 (58.7)	88 (81.5)	<0.0001
>12 months	1,248 (41.3)	20 (18.5)	
**Ever seen a patient less than 12 months of age for an infant/toddler visit**:			
Yes	2,221 (73.5)	103 (95.4)	<0.0001
No or don't know	802 (26.5)	5 (4.6)	
**Typically see patients under 12 months of age**:			
Yes	483 (16.0)	71 (66.7)	<0.0001
No or don't know	2,540 (84.0)	37 (34.3)	
**Do not see patients** **≤12 months of age, but refer to a colleague who does:**			
Yes	1,296 (55.7)	7 (24.1)	<0.001
No	1,030 (44.3)	22 (75.9)	
**Actively discusses early childhood dental care with your patients:**			
Yes	2,598 (94.6)	96 (100.0)	0.019
No	149 (5.4)	0 (0.0)	
**Promotes early visits for infants and toddlers in your practice:**			
Yes	2,153 (78.4)	93 (96.9)	<0.0001
No	594 (21.6)	3 (3.1)	
**Use “knee-to-knee positioning” when examining infants and toddlers:**			
Yes	1,579 (60.1)	80 (87.0)	<0.0001
No	1,047 (39.9)	12 (13.0)	
**Important for a child to receive their first dental examination within the first 6 months of the eruption of the first tooth, or by 1 year of age:**			
Agree	1,233 (49.4)	80 (89.9)	<0.0001
Somewhat agree	579 (23.2)	3 (3.4)	
Neither agree nor disagree	352 (14.1)	3 (3.4)	
Somewhat disagree	216 (8.7)	2 (2.3)	
Disagree	115 (4.6)	1 (1.0)	
**Confident in my ability to perform a dental examination on an infant:**			
Agree	1,333 (53.4)	86 (96.6)	<0.0001
Somewhat agree	700 (28.1)	1 (1.1)	
Neither agree nor disagree	249 (10.0)	2 (2.3)	
Somewhat disagree	158 (6.3)	0 (0.0)	
Disagree	55 (2.2)	0 (0.0)	
**Confident in my ability to perform a dental examination on a toddler:**			
Agree	1,642 (65.8)	86 (96.6)	<0.0001
Somewhat agree	602 (24.1)	2 (2.3)	
Neither agree nor disagree	137 (5.5)	1 (1.1)	
Somewhat disagree	77 (3.1)	0 (0.0)	
Disagree	37 (1.5)	0 (0.0)	
**Staff actively encourages infant and toddler dental care:**			
Agree	683 (27.4)	75 (84.3)	<0.0001
Somewhat agree	683 (27.4)	7 (7.9)	
Neither agree nor disagree	709 (28.4)	5 (5.6)	
Somewhat disagree	274 (11.0)	1 (1.1)	
Disagree	146 (5.8)	1 (1.1)	
**Staff is comfortable dealing with infants and toddlers in our dental practice:**			
Agree	943 (37.8)	82 (92.1)	<0.0001
Somewhat agree	873 (35.0)	5 (5.7)	
Neither agree nor disagree	417 (16.7)	1 (1.1)	
Somewhat disagree	193 (7.7)	1 (1.1)	
Disagree	69 (2.8)	0 (0.0)	
**Would like to receive additional training on how to incorporate early childhood care into my practice:**			
Agree	625 (25.1)	11 (12.4)	<0.0001
Somewhat agree	785 (31.5)	8 (9.0)	
Neither agree nor disagree	608 (24.4)	21 (23.6)	
Somewhat disagree	234 (9.3)	7 (7.9)	
Disagree	243 (9.7)	42 (47.1)	
**Aware of CDA's position of the first dental visit:**			
Yes	1,552 (63.7)	82 (97.6)	<0.0001
No	886 (36.3)	2 (2.4)	

Variables found to be significantly associated with recommending first visits ≤ 12 months of age were grouped into four different themes, and were analyzed using multiple logistic regression. The four themed models included dentists' characteristics, behaviors, barriers encountered, and awareness of dental organizations' position on the first visit. The first model (dentists' characteristics; data not shown) included five covariates, and revealed that years in practice (*p* < 0.0001), location in Central Canada (*p* < 0.0001), female gender (*p* < 0.0001), type of dentist/pediatric dentists (*p* < 0.0001), and working in solo private practices (*p* < 0.001) were all significantly associated with recommendations of first visits by 12 months.

The second model (dentists' behaviors; data not shown) included 12 variables. Nine out of the 12 variables were significantly associated with recommendations of first visits by 12 months. This included if dentists typically saw patients ≤ 12 months; if they promoted early visits; used “knee-to-knee positioning”; felt that parents understood the importance of a child's first visit; if dentists felt it was important for a child to receive their first dental examination within 6 months of the eruption of the first tooth, or by age one; if they felt confident to perform infant and/or toddler examinations; if staff encouraged infant and toddler dental care (*p* < 0.0001); and if staff felt comfortable dealing with infants and/or toddlers (*p* < 0.01).

The third model (barriers encountered; data not shown) included nine variables. The variables that were significantly associated with recommendations of first visits by 12 months included if the dentist did not think it was necessary to see a child by 1 year of age (*p* < 0.0001), if dentists did not know not know how to treat children (*p* < 0.05), if dentists were never taught how to treat children (*p* < 0.05), if dentists were too busy to treat children (*p* < 0.05), if dentists were uncomfortable seeing uncooperative children (*p* < 0.0001), and if dentists thought that few parents saw the first visit as a priority (*p* < 0.0001).

The fourth model (awareness of recommendations by dental organizations; data not shown) included two variables. The age dentists believed North American dental organizations recommended for first visits, and awareness of the CDA's position on first dental visits were both significantly associated with recommendations of first visits by 12 months (*p* < 0.001).

One final multiple logistic regression model was constructed using forward selection (Table 6). This included those variables that were significant in exploratory themes one (dentists' characteristics), two (dentists' behaviors), and four (awareness of recommendations), along with the top three significant barriers from the third theme. Results revealed that those who practiced in Central Canada were 1.83 (95% CI: 1.44, 2.32) times more likely to recommend first visits by age one than those located in Western Canada. The odds ratio of general dentists recommending first visit by 12 months was reduced by 61% compared to pediatric dentists (95% CI: 0.16, 0.91). Dentists who typically saw a patient ≤ 12 months were 3.41 times more likely to recommend first visits by 12 months (95% CI: 2.41, 4.83). Participants who felt it was important to have first dental visits within 6 months of eruption of the first tooth, or by age one, were 19.3 times more likely to recommend first visits by 12 months of age (95% CI: 8.2, 45.71). If their staff actively encouraged infant and toddler dental care, dentists were 1.76 times more likely to recommend first visit by 12 months (95% CI: 1.34, 2.31). Participants who correctly knew what age dental organizations in North America recommended first visit were 5.28 times more likely to recommend first visit by 12 months (95% CI: 4.13, 6.76).

**Table 6:** Multi-predictor regression model with participant characteristics, behaviors, and awareness with and without barriers for recommending first visits ≤ 12 months.

**Variable**	**Regression coefficient**	**Odds ratios**	**95% CI for odds ratios**	***p*-Value**
**With barriers**				
Intercept	−0.89	–	–	–
Years in practice	0.03	1.03	1.02, 1.04	<0.0001
Central Canada region^a^	0.60	1.83	1.44, 2.32	<0.0001
Eastern Canada region^a^	0.03	1.02	0.72, 1.48	0.87
Gender^b^	−0.18	0.84	0.66, 1.05	0.13
Type of dentist^c^	−0.95	0.39	0.16, 0.91	0.03
Group private practice^d^	−0.2	0.82	0.65, 1.03	0.10
Non-private practice^d^	−1.31	0.27	0.16, 0.47	<0.0001
Typically see a patient ≤ 12 months^e^	1.23	3.41	2.41, 4.83	<0.0001
Promotes early visits^e^	0.98	2.66	1.85, 3.82	<0.0001
Use “knee-to-knee” positioning^e^	0.36	1.44	1.13, 1.83	0.003
Parents understand importance of a child's first dental visit^e^	−0.35	0.71	0.54, 0.93	0.015
Important to have first dental visit 6 months of eruption of the first tooth, or by age 1^e^	2.96	19.32	8.2, 45.71	<0.0001
Confident to perform infant exam^e^	0.77	2.15	1.44, 3.23	0.0002
Confident to perform toddler exam^e^	−0.84	0.43	0.26, 0.72	0.0014
Front office staff encourages infant and toddler dental care^e^	0.57	1.76	1.34, 2.31	<0.0001
Staff is comfortable dealing with infants and toddlers in our dental practice^e^	−0.4	0.67	0.49, 0.92	0.014
Is not necessary^e^	−1.23	0.29	0.18, 0.49	<0.0001
Uncomfortable seeing un-cooperative children^e^	−0.49	0.61	0.43, 0.87	0.007
Few parents see it as a priority^e^	−0.40	0.67	0.52, 0.85	0.0013
Age dentists believe dental organizations in North America recommend first visit	1.66	5.28	4.13, 6.76	<0.0001
Aware of CDA's position of first dental visit^e^	0.38	1.47	1.15, 1.88	0.002
**Without barriers**				
Intercept	−3.02	–	–	–
Years in practice	0.03	1.03	1.02, 1.04	<0.0001
Central Canada region^a^	0.64	1.89	1.5, 2.4	<0.0001
Eastern Canada region^a^	0.024	1.02	0.72, 1.47	0.9
Gender^b^	−0.23	0.79	0.63, 0.99	0.045
Type of dentist^c^	−0.93	0.4	0.17, 0.9	0.03
Group private practice^d^	−0.24	0.79	0.62, 0.99	0.04
Non-private practice^d^	−1.39	0.25	0.15, 0.43	<0.0001
Typically see a patient ≤ 12 months^e^	1.60	4.78	3.46, 6.6	<0.0001
Promotes early visits^e^	1.04	2.83	1.98, 4.06	<0.0001
Uses “knee-to-knee” positioning^e^	0.40	1.5	1.18, 1.9	0.0008
Parents understand importance of a child's first dental visit^e^	−0.33	0.72	0.55, 0.94	0.017
Important to have first dental visit 6 months of eruption of the first tooth, or by age 1^e^	3.16	23.6	10.1, 55.2	<0.0001
Confident to perform infant exam^e^	0.75	2.12	1.4, 3.2	0.0002
Confident to perform toddler exam^e^	−0.79	0.46	0.27, 0.76	0.0024
Front office staff encourages infant and toddler dental care^e^	0.60	1.84	1.4, 2.4	0.0001
Staff is comfortable dealing with infants and toddlers in our dental practice^e^	−0.35	0.7	0.51, 0.96	0.03
Age dentists believe dental organizations in North America recommend first visit	1.7	5.41	4.25, 6.9	<0.0001
Aware of CDA's position of first dental visit^e^	0.39	1.48	1.16, 1.88	0.0017

Reference = ^a^Western Canada, ^b^Female, ^c^Pediatric dentist, ^d^solo private practice, ^e^no.

The second part of the final analyses also excluded barrier variables from the forward regression model (Table 6). When barrier variables were excluded, gender and group private practice became significant (*p* < 0.05). All other significant variables remained the same as in the analyses that included the barrier variables.

## Discussion

Dental organizations have been promoting first visits by age one for many years. As mentioned above, the first official North American policy statement on the concept of dental homes and first visits within the first year of life was published 35 years ago. There has been limited research regarding dentists' knowledge, attitudes, and behaviors on the first visit [7]. This study attempted to address this deficiency, and investigated Canadian dentists' views on the timing of a child's first dental visit, which is an important milestone that often occurs well beyond the recommended age of ≤ 12 months.

Research shows that there are benefits of early visits with the establishment of dental homes by meeting and identifying high-risk patients, and providing early preventive care [14]. There is growing recognition for the need to shift from rehabilitative treatments to oral health management and primary prevention, which can be best started with infants at the time of the eruption of the first tooth [6]. The CDA developed the “First Visit, First Tooth” campaign to raise public awareness and to educate dentists [15]. While all provincial dental associations follow the CDA's position on the timing of first dental visits, Manitoba and Prince Edward Island are the only two provinces that promote the Free First Visit (FFV) [16, 17]. The Manitoba Dental Association (MDA) started the FFV program in 2010 to promote access to care, and to encourage the idea of a dental visit within the first year of life [17].

When dentists were asked what age their dental organizations recommended patients to come for their first visit, most responded as soon as the first primary tooth erupts. However, when surveyed, dentists recommended a higher age for first visits. There is clearly a disconnect between the knowledge that dentists have with regard to the age of first visits and the age that they openly recommended. These findings are consistent with other studies [7, 8, 17–25]. Guidelines can provide information, but they do not always cause behaviors to change [21]. Earlier visit recommendations are preparing dentists to see children before their first birthday. It is encouraging that most practitioners in this study have seen children ≤ 12 months, but in reality, <20% of the dentists surveyed see one regularly.

With the introduction of the FFV in Manitoba, dentists appeared to be more aware of the recommended timing of first dental visits and early childhood oral health [21]. A study conducted in 2008 found that Manitoba dentists recommended a mean age of 24.8 ± 10.9 months for first visits. Following the introduction of the FFV program, which was launched by the MDA in 2010, a subsequent study found that dentists had begun recommending a younger age for first visits (mean 18.1 ± 10.0 months) [17]. A survey from 2013 showed that Manitoba dentists were recommending a mean age of 17.2 ± 10.6 months for first visits. The mean age may have dropped due to the promotion of earlier visits by the MDA, and the greater awareness of Manitoba dentists as a result.

A high number of Manitoba dentists have expressed beliefs that parents do not see the first visit as a priority, and that there is little demand for early visits [21]. This is a significant barrier, as parents need to be educated, informed, and engaged in their children's oral health. The first step for parents or caregivers should be to bring their child to the dentist within the first year of life [20, 21]. When parents acquire more education about the first visit, there should be an increase in requests for first visits, and dentists will have greater opportunities to provide services. Early childhood education programs can also improve dental care use, especially the use of preventive dental services among infants and toddlers at risk for dental disease [26]. While parental education about child's first dental visit is important, it is also crucial to take a closer look at other social determinants of health that may be at play. Reasons such as lack of transportation, financial constraints, having a sick child may not make dental visit a priority unless there is pain or infection. These underlying factors need to be addressed [27].

In this study, there were some deterrents that were identified for dentists not seeing infants and toddlers in their practice. Participants reported that they were uncomfortable examining children who were uncooperative and crying. Some felt that a first visit by 12 months was not necessary, which is a sentiment that is consistent with other studies [8, 17, 28, 29]. Dentists have requested additional training for seeing infants in the form of continuing education events, educational material and hands-on training [8, 21]. Research suggests that strategies such as professional education, journal articles, and advertising can help increase awareness for both providers and parents [21].

Female dentists recommended younger ages for first visits when compared to their male colleagues. This is consistent with prior research, where female clinicians have been more inclined to recommend first visits within the first year of life [21, 28]. Dentists who recommended first visits ≤ 12 months practiced for a shorter amount of time than dentist who recommended first visits >12 months. This suggests that the longer dentists practiced, the greater the age that was recommended for first visits to patients. This finding may be because dentists who have practiced for a shorter length of time may also have recently graduated from school and the importance of a child's first dental visit may now be part of the current curriculum. These findings are also consistent with previous studies [8, 19, 20, 28]. This study also showed that more dentists in non-private practices recommended a visit within the first year of life than those in solo or group practices. Greater awareness of the timing of first dental visits because of academic affiliations for non-private dentists working in hospital- or university-based settings could explain these results.

Due to the nature of their training, Canadian pediatric dentists in this study recommended earlier ages for first visits. Pediatric dentists knew the correct age to recommend first visits, used the “knee-to-knee positioning” to examine infants and toddlers, and their staff were more comfortable dealing with younger populations. These findings are characteristic of this group of professionals [7, 17]. A dental team should be trained in behavior management techniques since the staff is an extension of the dentist and are an integral part of in the line of communication with the child. A collaborative approach helps ensure that both the patient and the parent have a positive dental experience. All dental team members are encouraged to expand their skills and knowledge through dental literature, video presentations, and continuing education courses [30].

Key predictors for practitioners that recommended first visits within the first year of life included working in Central Canada, being female, being a pediatric dentist, working in solo private practices, and typically seeing patients ≤ 12 months. Dentists working in Central Canada may be more knowledgeable in infant oral health probably because these provinces are larger, have more pediatric dentists and more access to current and continuous dental education. Other predictors included promotion of early visits by practitioners, knowing the importance of first visits within the first year of life, belief that first visits are necessary, knowing the age dental organizations recommend, and having front office staff that encourage infant and toddler dental care. Dentists who use the “knee-to-knee positioning” technique, which is the recommended method of examining infants and toddlers, have tended to examine younger patient populations before their first birthday. Dentists who use ‘knee-to-knee' technique may also have had training in infant oral health care and this may account for their recommendation of a first visit within the first year of life [31, 32].

In the latter part of our last model, gender became a significant measure only when barriers were removed. These findings suggest important restrictions for male dentists with regard to early childhood visits. It is noted that the number of male respondents was greater than the number of female respondents in the original data set, and that trends in gender diversity in past dental graduation classes may also serve as a compounding factor. Group private practice also became significant measure in the last model. Practitioners in these types of practices may also have significant barriers in examining infants, and may rely on other providers in their practice to see children that come in.

Many, but not all, dental professional programs teach the recommended age for a first dental visit. One way to get through to dentists, especially general dentists, is to change what we teach. We must ensure that dental schools teach infant oral health, adhere to national guidelines, remove current barriers to education, and provide students with opportunities to see infants and toddlers in their undergraduate learning years [7, 21, 22, 28, 33]. This can be achieved through specialty clinics and community-based clinics, or by having dental students practice first visits on an infant of a volunteer parent.

First visits are also restricted by the limited number of pediatric dentists in Canada. As a majority of dental practitioners are general dentists, they will need to develop their skills in order to help fulfill the CDA's vision and position on the timing of the first dental visit. The CDA should consider targeting its educational campaigns to dentists in Eastern and Western Canada, male dentists, general dentists, and those in group private practices to better recommendations for dental visit within the first year of life. Future research will help determine the impact of campaigns for the first dental visits, and show whether this leads to a reduction in ECC and rates of dental surgery [17].

This study is not without limitations. While 3,232 dentists participated, the response rate was modest. Additionally, recall and response bias is possible. It is likely that those responding to the survey were most interested in the topic, and were already seeing younger patients. Thus, our findings may not be entirely representative of the average Canadian dentist. Also, the CDA survey was conducted in 2013 and this may not reflect the current opinion of Canadian dentists on child's first dental visit. Follow-up surveys to assess and compare current practices, attitudes, opinions and recommendations of Canadian dentists on a child's first dental visit are recommended. Strengths of this study include the fact that it is the first national survey of CDA members regarding timing of first dental visit, and there was a relatively large sample size.

## Conclusions

More than half of all dentists that participated in this survey did not recommend first dental visits by 12 months of age, even though this is the CDA's official position. Significant associations for recommendations of early first visits were seen for Central Canadian dentists, female dentists, pediatric dentists, and those working in solo private practices. Findings from this study can guide targeted educational campaigns for practicing dentists and those in training. This study serves as a baseline for future changes in dentists' knowledge, attitudes, and behaviors on first dental visits, and will hopefully be instrumental in children being seen at an earlier age.

## Data availability statement

The dataset used and analyzed during this study was provided by the Canadian Dental Association, who own the data. Requests to access the datasets should be directed at: reception@cda-adc.

## Ethics statement

The studies involving human participants were reviewed and approved by University of Manitoba Health Research Ethics Board. The patients/participants provided their written informed consent to participate in this study.

## Author contributions

HA-T led the analysis, interpretation of the data, and was a major contributor in writing the manuscript. RS created the study protocol, helped analyze and interpret data, and was a major contributor in writing the manuscript. RH, VL, and OO were significant contributors in the writing of the manuscript. All authors contributed to the article and approved the submitted version.

## Funding

Operating funds to support data analyses reported in this study were provided by the Dr. Gerald Niznick College of Dentistry Endowment Fund, University of Manitoba. Partial publication charges were provided by the Department of Preventive Dental Science, Dr. Gerald Niznick College of Dentistry, University of Manitoba.

## Conflict of interest

Author RS was employed by Shared Health Inc. The remaining authors declare that the research was conducted in the absence of any commercial or financial relationships that could be construed as a potential conflict of interest.

## Publisher's note

All claims expressed in this article are solely those of the authors and do not necessarily represent those of their affiliated organizations, or those of the publisher, the editors and the reviewers. Any product that may be evaluated in this article, or claim that may be made by its manufacturer, is not guaranteed or endorsed by the publisher.

## References

[1] El Tantawi M Folayan MO Mehaina M Vukovic A Castillo JL Gaffar BO. Prevalence and data availability of early childhood caries in 193 United Nations Countries, 2007-2017. Am J Public Health. (2018) 108:1066–72. 10.2105/AJPH.2018.304466 29927650 PMC6050821

[2] Pierce A Singh S Lee J Grant C Cruz de. Jesus V, Schroth RJ. The burden of early childhood caries in Canadian children and associated risk factors. Front Public Health. (2019) 7:328. 10.3389/fpubh.2019.00328 31781530 PMC6861386

[3] Schroth RJ Harrison RL Moffatt ME. Oral health of indigenous children and the influence of early childhood caries on childhood health and well-being. Pediatr Clin North Am. (2009) 56:1481–99. 10.1016/j.pcl.2009.09.010 19962032

[4] Ramos-Gomez FJ. A model for community-based pediatric oral heath: implementation of an infant oral care program. Int J Dent. (2014) 2014:156821. 10.1155/2014/156821 24587803 PMC3920860

[5] Rowan-Legg A Canadian Paediatric Society CPC. Oral health care for children - a call for action. Paediatr Child Health. (2013) 18:37–50. 10.1093/pch/18.1.37 24381493 PMC3680273

[6] Nowak AJ. Paradigm shift: Infant oral health care–primary prevention. J Dent. (2011) 39(Suppl 2):S49–55. 10.1016/j.jdent.2011.11.005 22101124

[7] Santos CL Douglass JM. Practices and opinions of pediatric and general dentists in Connecticut regarding the age 1 dental visit and dental care for children younger than 3 years old. Pediatr Dent. (2008) 30:348–51. 18767516

[8] Stijacic T Schroth RJ Lawrence HP. Are Manitoba dentists aware of the recommendation for a first visit to the dentist by age 1 year? J Can Dent Assoc. (2008) 74:903. 19126358

[9] Darmawikarta D Chen Y Carsley S Birken CS Parkin PC Schroth RJ. Factors associated with dental care utilization in early childhood. Pediatrics. (2014) 133:e1594–600. 10.1542/peds.2013-3725 24799544

[10] Savage MF Lee JY Kotch JB Vann WF Jr. Early preventive dental visits: effects on subsequent utilization and costs. Pediatrics. (2004) 114:e418–23. 10.1542/peds.2003-0469-F 15466066

[11] Examination P. Anticipatory guidance/counseling, and oral treatment for infants, children, and adolescents. Pediatr Dent. (2018) 40:194–204. 32074888

[12] Schroth RJ Christensen J Morris M Gregory P Mittermuller BA Rockman-Greenberg C. The influence of prenatal vitamin D supplementation on dental caries in infants. J Can Dent Assoc. (2020) 86:k13. 33326371

[13] Schroth R Wilson A Prowse S Edwards J Gojda J Sarson J. Looking back to move forward: Understanding service provider, parent, and caregiver views on early childhood oral health promotion in Manitoba, Canada. Can J Dent Hyg. (2014) 48:99−108.

[14] Hale KJ American Academy of Pediatrics Section on Pediatric D. Oral health risk assessment timing and establishment of the dental home. Pediatrics. (2003) 111:1113–6. 10.1542/peds.111.5.1113 12728101

[15] Canadian Dental Associan. First visit, first tooth Ottawa: Canadian Dental Association. (2017). Available online at: https://www.firstvisitfirsttooth.ca (accessed June 1, 2021).

[16] Muttart M. Island Toddlers' First Dental Visit Now Free. https://www.google.com/search?client=firefox-b-d&q=Halifax+Nova+Scotia,+Canada&stick=H4sIAAAAAAAAAONgVuLUz9U3MDbLNihexCrjkZiTmZZYoaPgl1-WqBCcnF-Smaij4JyYl5iSCABShYkiLAAAAA&sa=X&ved=2ahUKEwjZztT2zar5AhWPFlkFHS_yD1UQmxMoAHoECFQQAg Halifax, NS: Saltwire (2015).

[17] Schroth RJ Guenther K Ndayisenga S Marchessault G Prowse S Hai-Santiago K. Dentists' perspectives on the Manitoba Dental Association's free first visit program. J Can Dent Assoc. (2015) 81:f21. 10.1111/jphd.12137 26679335

[18] Brickhouse TH Unkel JH Kancitis I Best AM Davis RD. Infant oral health care: a survey of general dentists, pediatric dentists, and pediatricians in Virginia. Pediatr Dent. (2008) 30:147–53. 18481580

[19] Malcheff S Pink TC Sohn W Inglehart MR Briskie D. Infant oral health examinations: pediatric dentists' professional behavior and attitudes. Pediatr Dent. (2009) 31:202–9. 10.1308/135576109789389388 19552224

[20] Bubna S Perez-Spiess S Cernigliaro J Julliard K. Infant oral health care: beliefs and practices of American Academy of Pediatric Dentistry members. Pediatr Dent. (2012) 34:203–9. 22795152

[21] Schroth RJ Yaffe AB Edwards JM Hai-Santiago K Ellis M Moffatt ME. Dentist's views on a province-wide campaign promoting early dental visits for young children. J Can Dent Assoc. (2013) 79:d138. 24598319

[22] Schroth RJ Quinonez RB Yaffe AB Bertone MF Hardwick FK Harrison RL. What are canadian dental professional students taught about infant, toddler and prenatal oral health. J Can Dent Assoc. (2015) 81:f15. 26352522

[23] Hussein AS Schroth RJ Abu-Hassan MI. General dental practitioners' views on early childhood caries and timing of the first dental visit in Selangor, Malaysia. Asia Pac J Public Health. (2015) 27:NP2326–38. 10.1177/1010539513475645 23420056

[24] Mika A Mitus-Kenig M Zeglen A Drapella-Gasior D Rutkowska K Josko-Ochojska J. The child's first dental visit. Age, reasons, oral health status and dental treatment needs among children in Southern Poland. Eur J Paediatr Dent. (2018) 19:265–70. 10.23804/ejpd.2018.19.04.3 30567441

[25] Djokic J Bowen A Dooa J Kahatab R Kumagai T McKee K. Knowledge, attitudes and behaviour regarding the infant oral health visit: are dentists in Ireland aware of the recommendation for a first visit to the dentist by age 1 year? Eur Arch Paediatr Dent. (2018) 20:56–72. 10.1007/s40368-018-0386-0 30378001

[26] Burgette JM Preisser JS Jr Weinberger M King RS Lee JY Rozier RG. Impact of early head start in North Carolina on dental care use among children younger than 3 years. Am J Public Health. (2017) 107:614–20. 10.2105/AJPH.2016.303621 28207343 PMC5343690

[27] Olatosi OO Onyejaka NK Oyapero A Ashaolu JF Abe A. Age and reasons for first dental visit among children in Lagos, Nigeria. Niger Postgrad Med J. (2019) 26:158–63. 10.4103/npmj.npmj_60_19 31441453

[28] Wolfe JD Weber-Gasparoni K Kanellis MJ Qian F. Survey of Iowa general dentists regarding the age 1 dental visit. Pediatr Dent. (2006) 28:325–31. 16903440

[29] Garg S Rubin T Jasek J Weinstein J Helburn L Kaye K. How willing are dentists to treat young children?: a survey of dentists affiliated with Medicaid managed care in New York City, 2010. J Am Dent Assoc. (2013) 144:416–25. 10.14219/jada.archive.2013.0135 23543696

[30] Behavior guidance for the pediatric dental patient. Pediatr Dent. (2018) 40:254–67. 32074897

[31] Anderson R. CE Showcase: Knee-to-Knee Examination with Dr. Ross Anderson. Ottawa, ON: Canadian Dental Association (2016).

[32] Hardwick F. Point of Care. How do I perform a first dental visit for an infant or toddler? J Can Dent Assoc. (2009) 75:577–8. 19840497

[33] Nascimento MM Mugayar L Tomar SL Garvan CW Catalanotto FA Behar-Horenstein LS. The impact of an infant oral health program on dental students' knowledge and attitudes. J Dent Educ. (2016) 80:1328–36. 10.1002/j.0022-0337.2016.80.11.tb06218.x 27803205

